# Doula support among brazilian women who attended the senses of birth health education intervention – a cross sectional analysis

**DOI:** 10.1186/s12884-022-05069-0

**Published:** 2022-10-12

**Authors:** Luísa M M Fernandes, Kathryn E Mishkin, Sônia Lansky

**Affiliations:** 1 Instituto René Rachou, Fundação Oswaldo Cruz (FIOCRUZ), Minas Gerais, Brazil; 2 Independent researcher, Washington, DC USA; 3 Department of Health, City Hall, Belo Horizonte, Minas Gerais Brazil

**Keywords:** Doula, Brazil, Cesarean, Evidence-based practice, Maternal mortality, Vaginal birth, Normal birth

## Abstract

**Background:**

While maternal health is a priority in international goals, maternal health outcomes remain poor in many regions of the world. In Brazil, maternal mortality has decreased over the past decades, but the country’s maternal mortality ratio is higher than over half of all countries at 59 deaths per 100,000 live births. The Brazilian maternal health care model facilitates high rates of medical interventions during labor and childbirth; 56% of births are by cesarean birth. Doula support is considered a potential strategy to reduce medically unnecessary interventions during childbirth that contribute to maternal mortality.

**Methods:**

The cross-sectional study analyses associations with use of doula support and normal birth among Brazilian women who participated in a health education intervention named the Senses of Birth (SoB). The SoB intervention, implemented in five cities from 2015 to 2017, was developed to educate about normal birth and to evidence-based practices (EBP) reduce medically in childbirth. Chi-Square tests were performed to identify the relationship between doula support during childbirth and sociodemographic characteristics, childbirth information, perceived knowledge, and use of EBPs during labor. Logistic regression was performed to identify associations in adjusted analysis.

**Results:**

Controlling for covariates, doula support was associated with vaginal delivery (OR 2.47, 95% CI: 1.37–4.45.) Findings also suggest that women who had doula support were more likely to use non-pharmacological pain relief methods during labor (OR 9.68, 95% CI: 2.67–34.61), deliver in a public hospital (OR 2.02, 95% CI: 1.09–3.72), and be low and mid-level income compared to women with high income.

**Conclusion:**

This study’s findings suggest that doula support is significantly associated with vaginal birth. The results may be useful for advocating for changes to the childbirth care model in Brazil. Incorporating EBPs, such as doula support, for all women who desire may improve maternal and child outcomes.

## Background

Maternal health is a key priority for global health. One quarter of a decade since women’s rights were declared human rights at the United Nations Declaration and Platform for Action, improving women’s health is still considered a primary objective for the global health community [1]. Several of the World Health Organization (WHO) Sustainable Development Goals (SDG), including SDG Goal 3, which seeks to ensure healthy lives and promote well-being for all at all age; and SGD Goal 5, which addresses gender equality and is focused on ensuring universal access to sexual and reproductive health and reproductive rights [2].

In spite of the dedication of the global health community, several maternal and child health indicators suggest that there are still gaps in access to and use of maternal health care services and resources [3]. According to the WHO, while the global maternal mortality ratio declined by 38% between 2000 and 2017, roughly 295,000 women and girls died in 2017 during pregnancy or childbirth; most of these deaths were preventable [4]. Hemorrhage, sepsis, pre-eclampsia and eclampsia, complications in delivery, and unsafe abortion are the most common causes of maternal mortality[5, 6].

Non-medically indicated cesarean birth is a leading driver of maternal mortality and morbidity. Maternal mortality occurs two to seven times more frequently among women who had a cesarean birth when compared to vaginal birth [7, 8]. A global population study analyzing births in 24 countries found that cesarean birth was associated with increased risk of serious complications including hemorrhage and issues with analgesia [9]. Birth by cesarean section increases the risk of complications not only immediately after the surgery, but also in subsequent pregnancies and childbirth [9]. Costs associated with cesarean birth are also higher than vaginal birth [10], requiring longer hospital stays post-delivery and more costly medical procedures [11].

As is the case in Latin America in general [12], maternal death is associated with cesarean birth in Brazil [8]. Brazil implemented a national health policy named the “Stork Network” in 2011 to support women’s reproductive health through pregnancy, childbirth, and the postpartum period and ensure maternal health, newborn health, and good infant growth [13]. Still, Brazil has one of the highest cesarean birth rates in the world at 56% [14]. A 2016 national study found that cesarean birth accounted for all deaths from complications of anesthesia and was associated with significant risk of death from postpartum hemorrhage [8]. Research focused on one region in Brazil showed that cesarean birth was associated with lower use of evidence-based practices (EBP) including immediate skin-to-skin contact and first-hour breastfeeding; and worse health outcomes including higher frequency of newborns referred to neonatal intensive care units, and more postpartum infections [15]. Despite efforts to improve maternal care, Brazil’s maternal mortality ratio stands at 59.1 deaths per 100,000 live births [16, 17].

Continuous support in labor and delivery, particularly from a doula, is associated with lower likelihood of cesarean birth [18]. Doulas are lay people trained in labor support who provide continuous support throughout pregnancy, childbirth, and the postpartum period [19]. Findings from a 2019 Cochrane Review and other studies suggest that women who have doula support develop close bonds with their doulas during childbirth, which supports mothers’ physical and mental health in the maternal period [18, 20].

In addition to reducing the likelihood of cesarean birth, the literature shows that women who have doula support are more likely to have a shorter delivery length, to use non-medical techniques to induce labor [19, 21–23], and to be satisfied with their birth [18, 24, 25]. After delivery, women who had doula support are also more likely to breastfeed, report good self-esteem, and believe they are able to care for their infants compared to those who did not use a doula [26]. Prenatal doula use is also associated with a 40% lower odds of preterm birth [26–28].

To improve quality of birth experience in 2005, Brazil enacted a national law to ensure women the right to have a companion of their choice in childbirth in every hospital or birth center at no cost to the patient [29]. A companion of choice can include any family member or friend. Studies show this law supported women to have companions in labor and delivery: the Birth in Brazil national study demonstrated that only 18.8% of women had continuous companionship during delivery and childbirth in 2012 [32], but this figure had increased to 163% of women having continuous companionship by 2017 [33].

To further advance doula support, in 2006, the Ministry of Health and National Policy on Integrative and Complementary Practices included doulas as Traditional Medicine and Alternative and Complementary Medicine professionals, and in 2013, doulas were provided an occupational title, which formalized and legitimized their role as support providers in the eyes of the government [30]. The occupational title defines a minimum scope of practice, however it does not regulates the profession or relates to training requirements Currently two different types of doula models can be observed in Brazil: private and public. Private doulas may be hired by patients at an out-of-pocket cost to provide their services in private hospitals. Since the profession is not regulated, charges frequently range from USD $200 to $600. Public doulas, also known as community doulas, are volunteers who provide services for free to women delivering at public hospitals within Brazil’s Public Health System (SUS). The first public volunteer doula program in the country was established in Belo Horizonte City at Sofia Feldman’s Hospital [31]. Since its inception, this community model has been slowly implemented in other public and philanthropic hospitals in the country. However, research on doula use in Brazil is limited and no study has been published exploring associations between doulas and cesarean birth.

Because doula support is associated with lower rates of cesarean birth in global contexts, it is hypothesized that doulas may be an important strategy to reducing medically unnecessary cesarean births in Brazil. This paper aims to identify the association between doula support during childbirth and cesarean births among women who participated in a health education intervention named Senses of Birth.

## Methods

This cross-sectional study investigates the impact of doula support during childbirth among Brazilian women who previously participated in a health education intervention named the Senses of Birth (SoB).

### The senses of birth intervention

The SoB intervention was developed to promote normal birth in Brazil and to reduce medically unnecessary interventions in childbirth. This international grant-funded public educational intervention was implemented in five large population cities in Brazil from 2015 to 2017 to engage the local community and disseminate information regarding evidence-based practices in childbirth [32]. The interactive intervention led participants through the stages of pregnancy, childbirth, and the postpartum period and demonstratedEBPs and valuing the physiology of normal birth [32]. All visitors were invited to walk through a scenario, first as a pregnant woman, and later as a newborn baby. Myths and facts about prenatal health, labor and delivery, and the postpartum period were discussed using theater, sensorial experience, and a video panel discussion [32].

### Data and sample

This paper uses data collected as part of the SoB project research analysis. All women provided written informed consent prior to answering the post-intervention survey and the follow-up survey. All participants were invited to complete a paper-based self-administered structured questionnaire after participation at SoB containing sociodemographic characteristics and questions about perceived knowledge related to normal birth, cesarean sections, and EBPs. All 1,287 pregnant women over the age of 18 years who answered the post-intervention survey were invited to complete an online self-administered structured questionnaire after childbirth.

The follow-up survey included questions related to the most recent labor and childbirth experience, use of the EBPs during childbirth, and memory and influence of the educational intervention on their childbirth experience. Five hundred and fifty-five women answered the follow-up questionnaire (43% of the original sample) between June 2015 and April 2016. Women who did not respond to the survey after three email invites and three phone calls were eliminated.

### Variables

Use of doula support during childbirth data was collected through a closed-ended question regarding the professional(s) present during childbirth. The answer options were: doula, obstetrician, midwife, obstetric nurse, pediatrician, no health professional present, and others. The participant could choose as many options as applicable. A dichotomous variable to indicate doula support (doula support vs. no doula support) was created.

Sociodemographic characteristics included age (19 to 34 years old vs. ≥ 35 years old), race (white vs. non-white (Black, Asian, and indigenous)), education level (< 12 years, ≥ 12 years), private health insurance (yes, no), and income (very poor, poor, moderate, and wealthy). In Brazil, services rendered through the national public health system (known as the Unified Health System or SUS) are available to all residents and nonresidents without out-of-pocket charges or co-payments^41^. Some residents (ranging from 22 to 25% of the population) also have additional coverage through private insurance, commonly financed by employers. Income was measured using the monthly family earnings relative to the country minimum wage. The income levels included Very Poor = < 2 minimum wage, Poor = 2 to < 5 minimum wage, Moderate = 5 to < 10 minimum wage, Wealthy ≥ 10 minimum wage One minimum wage at the time of the intervention was approximately R$788.00, which was USD $224.14. Childbirth experience included questions related to pregnancy: first pregnancy (yes vs. no), type of delivery (vaginal vs. cesarean), type of hospital of delivery (SUS- public hospital vs. private hospital) indicating the mode of finance. “SUS” refers to public or non-profit hospitals integrated with the public health system, funded by the government, without any direct payment of patients for any care. “Private” refers to for- profit or non-profit hospitals not funded by the government, healthcare paid by privately-owned health insurance or direct or out of pocket payment by the patients.

The EBPs described in this paper were identified through a review of the literature [15, 32]. They were incorporated into the education provided through SoB and included: midwife care during childbirth (yes vs. no); freedom of mobility during labor (yes vs. no) characterized as the ability to walk, dance, and crouch; position during delivery (lithotomy position vs. no lithotomy position) where non-lithotomy position was characterized as any position other than supine (traditional gynecological position) including kneeling, semi-sitting with support, or sitting upright; and use of non-pharmacological methods for pain relief (yes vs. no), including massage, use of a birth ball, shower, bathtub, electrodes (TENS), music, meditation, and breathing techniques.

Perceived knowledge about normal birth, cesarean section delivery, and EBPs was measured through questions using a Likert scale (1 to 3 – low and 3.01 to 5 high). The different questions were combined using a factor analysis to create a domain knowledge variable for normal birth, cesarean section delivery, and EBPs. The method used for the combined variable has been described in a previous publication.

### Statistical analysis

Chi-Square tests were performed to identify the relationship between doula support during childbirth and type of delivery, sociodemographic characteristics, childbirth information, perceived knowledge, and use of EBPs during labor. Logistic regression analyses were performed to identify association in an adjusted analysis and associations were considered statistically significant with P-value ≤ 0.05. The statistical program IBM SPSS Statistics 24R was used for the data analysis.

Two regression models were constructed to identify association with doula support during childbirth compared to no doula support: Model 1 - all women participating in the study; Model 2 – subsample of women who had vaginal birth. All variables that obtained a p-value equal to or less than 0.20 in the bivariate analysis were included in the logistic regression model. The magnitude of the association in the logistic regression models was evaluated through odds ratio (OR) and their respective confidence intervals at 95%. The quality of the fit of the model was assessed by the Hosmer-Lemeshowe test and the explanatory power of the model was assessed by the Nagelkerke pseudo-R².

### Ethical statement

This study is part of the research project named “Senses of Birth: Effects of the interactive exhibition in the perception changes on labor and childbirth.“ The Federal University approved the study at Minas Gerais IRB (COEP/UFMG, 934.472) and the University at Albany Institutional Review Board approved the study (Protocol Number: 18-X-209-01). All participants provided written informed consent prior to participation.

## Results

The majority of the participants were younger than 34 years (87.0%), white (54.8%), highly educated (80.8%), had a family income higher than 5 minimum wages (mid to high income) (52.2%), and had private health insurance (78.8%) (Table 1). 26% of women received doula support during childbirth.

**Table 1:** Characteristics of women who participated in the SoB intervention. Brazil 2015–2016 (n = 542)

Characteristics^1^	Received Doula Support	Did not Receive Doula Support	P-value
	**N (%)** 146 (26.9)	**N (%)** 396 (73.1)	
**Age**			
19–34 years	127 (87.0)	318 (81.1)	0.110
≥ 35 years	19 (13.0)	74 (18.9)	
TOTAL	146	392	
**Education**			
< 12 years	28 (19.2)	101 (25.7)	0.115
≥ 12 years	118 (80.8)	292 (74.3)	
TOTAL	146	393	
**Income** ^**2**^			
Very Poor	24 (17.6)	75 (20.2)	0.060ˆ
Poor	41 (30.1)	125 (33.8)	
Moderate	30 (22.1)	100 (27.0)	
Wealthy	41 (30.1)	70 (18.9)	
TOTAL	136	370	
**Race**			
White	80 (54.8)	170 (43.3)	0.017*
Black and Others	66 (45.2)	223 (56.7)	
TOTAL	146	393	
**Private Health Insurance**			
Yes	115 (78.8)	310 (78.7)	0.983
No	31 (21.2)	84 (31.3)	
TOTAL	146	394	
**Type of Hospital**			
Public (SUS^3)^	76 (52.4)	119 (30.1)	0.000**
Private	69 (47.6)	277 (69.9)	
TOTAL	145	396	
**Type of Birth**			
Vaginal	118 (80.8)	178 (44.9)	0.000**
Cesarean	28 (19.2)	218 (55.1)	
TOTAL	146	396	
**First Pregnancy**			
Yes	58 (45.3)	175 (50.0)	0.364
No	70 (54.7)	175 (50.0)	
TOTAL	128	350	
**Received Midwife Care**			
Yes	97 (66.4)	197 (49.7)	0.004*
No	49 (33.6)	199 (50.3)	
TOTAL	143	387	
**Freedom of mobility during labor**			
Yes	113 (80.7)	126 (45.0)	0.000**
No	27 (19.3)	154 (55.0)	
TOTAL	140	280	
**Position at delivery**			
Not Lithotomic	109 (77.9)	128 (45.7)	0.000**
Lithotomic	31 (22.1)	152 (54.3)	
TOTAL	140	280	
**Use of non-pharmacological pain relief methods**			
Yes (any)	136 (97.1)	171 (62.0)	0.000**
No (analgesia only)	4 (2.9)	105 (38.0)	
TOTAL	140	276	
**Normal Birth Knowledge After SoB**			
Low Knowledge	3 (2.1)	17 (4.3)	0.220
High Knowledge	143 (97.9)	379 (95.7)	
TOTAL	146	396	
**Cesarean Knowledge After SoB**			
Low Knowledge	9 (6.2)	27 (6.9)	0.785
High Knowledge	136 (93.8)	366 (93.1)	
TOTAL	145	393	
**EBP Knowledge After SoB**			
Low Knowledge	5 (3.5)	37 (9.4)	0.023*
High Knowledge	139 (96.5)	356 (90.6)	
TOTAL	144	393	

^1^Total varies dues to missing data for each variable

^2^Monthly Minimum Wage in 2015: R$788.00 = U$224.14

^3^SUS – Unified Health System

^P value ≤ 0.1; *P value ≤ 0.05; **P value ≤ 0.001.

### Bivariate analysis of doula support during childbirth

Women who received doula support in childbirth were more likely to be white (p = 0.17), have a vaginal birth (p < 0.001), have freedom of mobility during labor (p < 0.001), receive midwife care during childbirth (p = 0.004), use non-pharmacological pain relief methods (p < 0.001), deliver in a non-lithotomy position (p < 0.001), deliver in a public hospital (p < 0.001), and have high knowledge of EBP (p = 0.02) (Table 1). Income level approached significance (p = 0.06).

### Logistic regression analysis to identify factors associated with doula support during childbirth among all women

Because doula support during childbirth was identified as a factor associated with vaginal delivery in the bivariate analysis, a logistic regression model was performed to identify other factors associated with doula support and to adjust for covariates. Results of analysis of all women (Model 1) showed that women who had doula support during childbirth were more likely to have low (OR 2.13, 95% CI: 1.02–4.41) and mid-level income (OR 2.08, 95% CI: 1.01–4.27) compared to women with higher income; deliver in a public hospital (OR 2.02, 95% CI: 1.09–3.72); deliver vaginally (OR 2.47, 95% CI: 1.37–4.45); and use non-pharmacological pain relief methods during labor (OR 9.68, 95% CI: 2.67–34.61) (Table 2).

**Table 2:** Associations with doula support among women who participated in the SoB intervention by mode of delivery

	Model 1 - All Women^2^			Model 2 - Women who delivered vaginally^2^		
	**Total N = 363** ^**1**^			**Total N = 233** ^**1**^		
**Characteristics**	**OR**	**CI 95%**	**Wald**	**OR**	**CI 95%**	**Wald**
			**(P-value)**			**(P-value)**
**Age**						
19–34 years	1.58	0.77–3.23	1.57 (0.21)	1.43	0.62–3.28	0.73 (0.39)
≥ 35 years	1.00			1.00		
**Education**						
< 12 years	1.00			1.00		
≥ 12 years	1.21	0.56–2.62	0.24 (0.61)	2.048	0.82–5.10	2.36 (0.12)
**Income³**						
Very Poor	1.83	0.68–4.91	1.44 (0.23)	1.04	0.43–4.48	0.32 (0.57)
Poor	2.12	1.02–4.41	4.13 (0.04)	1.96	0.83–4.64	2.39 (0.12)
Moderate	2.08	1.01–4.27	4.02 (0.04)	2.03	0.85–4.82	2.59 (0.10)
Wealthy	1.00			1.00		
**Race**						
White	1.60	0.93–2.77	2.93 (0.08)	1.58	0.81–3.05	1.84 (0.17)
Black and Others	1.00			1.00		
**Type of Hospital**						
Public (SUS^3^)	2.02	1.09–3.72	5.06 (0.02)	2.18	1.04–4.54	4.32 (0.03)
Private	1.00			1.00		
**Type of Birth**						
Vaginal	2.47	1.37–4.45	9.09 (0.00)	-	-	-
Cesarean	1.00			-	-	-
**Midwife Care**						
Yes	1.02	0.561–1.87	0.01 (0.93)	1.07	0.51–2.23	0.03 (0.84)
No	1.00			1.00		
**Freedom of mobility during labor**						
Yes	1.60	0.80–3.21	1.78 (0.18)	1.51	0.68–3.32	1.04 (0.30)
No	1.00			1.00		
**Position at delivery**						
Not Lithotomic	1.37	0.68–2.75	0.80 (0.36)	2.08	0.93–4.62	3.26 (0.07)
Lithotomic	1.00			1.00		
**Non-pharmacological pain relief methods**						
Yes (any)	9.62	2.67–34.61	12.003 (0.001)	7.75	1.61–37.21	6.55 (0.01)
No (analgesia only)	1.00			1.00		
**Normal Birth Knowledg After SoB**						
Low Knowledge	1.71	0.32–9.10	0.40 (0.52)	2.17	0.20–22.63	0.42 (0.51)
High Knowledge	1.00			1.00		
**EBP Knowledge After SoB**						
Low Knowledge	1.86	0.51–6.73	0.90 (0.34)	2.51	1.04–4.54	1.39 (0.23)
High Knowledge	1.00			1.00		

^1^Total varies dues to missing data for each variable

² Logistic Regressions with Doula support (impact) as the reference variable.

³Monthly Minimum Wage in 2015: R$788.00 = U$224.14.

All Women: Hosmer and Lemeshow Test X²=9.722; df = 8; P-value = 0.285. R² de Nagelkerke = 0,348.

Vaginal Birth Only: Hosmer and Lemeshow Test X²=4.349; df = 8; P-value = 0.824. R² de Nagelkerke = 0,320.

Missing: Listwise deletion.

### Logistic regression analysis to identify factors associated with doula support during childbirth among women who had a vaginal birth

An additional logistic regression model was performed to identify factors associated with doula support only among women who had a vaginal birth. (Model 2): women who had a vaginal birth and received doula support during childbirth were more likely to deliver in a public hospital (OR 2.18, 95% CI: 1.04–4.54) and use non-pharmacological pain relief methods (OR 7.76, 95% CI: 1.61–37.21) (Table 2). Multicollinearity for income and education was tested, and no substantive changes in the results were observed when excluding either variable from the model.

## Discussion

The majority (82%) of SoB participants had a companion of choice with them through labor, delivery and postpartum [33]. This likely reflects the result of the long-standing advocacy to ensure the implementation of the 2005 law [29]. Doula support was significantly associated with vaginal delivery among women who attended the SoB intervention, consistent with the literature in other global contexts [22, 27]. Considering that risk of maternal mortality is higher among cesarean deliveries compared to vaginal deliveries [12], this study’s findings suggest that widespread national use of doula support could be an important strategy to increase vaginal birth and contribute to the reduction of maternal mortality in Brazil.

Only 27% of all women in this study reported having doula support during childbirth overall. While there is a dearth of information surrounding doula use worldwide, it appears that use of doula in this study’s sample was higher compared to other populations. In the United States, a 2013 Listening to Mothers population survey estimated that 6% of women received doula during labor and delivery [34]. Several factors are associated with low use of doulas including women’s lack of knowledge about doula support, doula workforce shortage, structural factors related to how doulas practice in the public and the private health systems, and inadequate integration of doulas within birth care teams, among others, contribute to the limited use of doulas [18, 28, 35, 36].

To the authors’ knowledge, no other Brazilian studies have analyzed doula support as distinguished from continuous support from other types of birth companions, including family members. While there is evidence that use of any form of continuous support is associated with satisfaction withchildbirth and protection from obstetric violence, which is the mistreatment of birthing people in the childbirth setting [14, 18, 21, 25], it has been suggested that doulas might provide a tailored approach to the patient because they are considered to be professionalsand may work with the clinical birth team [18, 26, 28]. This study performed additional analysis to identify the difference in impact between between support from a doula compared to continuous support from all types of birth companions in general, not specifically from a doula. Continuous support from any type of companion was not significantly associated with type of birth (p = 0.23. Having companionship during childbirth was likely not associated with type of delivery because women legally have the right to companionship [29] and by 2017, the majority of Brazilian women reported having a companion at delivery [33]. The current results support the hypothesis that usinga doula’s services in particular may decrease the frequency of medically unnecessary cesareans in Brazil. Additional studies regarding support provided by doulas and other birth companions should be completed to deepen our understanding of the importance of support for childbirth outcomes and experience.

The majority of women who received doula support had private health insurance and gave birth at the public hospital. We hypothesize that women participating at the SoB Intervention may have improved their knowledge about the obstetric model of care and learned that the public hospital system was more likely to support vaginal birth and humanized care.

The prevalence of cesarean births in this study’s sample was 46%, which, despite being high, is lower than the country’s average rate of 56%. The population of this study consisted predominantly of women who were highly educated, had high income, and had access to private health insurance, which suggests that they were predominantly a socioeconomic privileged group who could advocate for the use of EBP, including doula support. Further, while participants likely had lifelong exposure to the prevalent maternal health practice norms in the country, including high use of cesarean delivery, the authors acknowledge the participants may have been interested in the content of the SoB intervention, including the normalization of birth, since they chose to participate. Further, we acknowledge that women who choose to use doula support in childbirth may also be motivated to avoid unnecessary medical interventions including cesarean birth. While the study may not represent all Brazilian women, the findings suggest that education may be useful for changing norms.

## Conclusion

Overall, the findings from this study suggest that access to doula support during childbirth is limited. Only 27% of participants had doula support during childbirth., Consistent with the literature from other countries, this study’s finding suggest that Brazilian women who have doula support are more likely to have vaginal birth. Investments to improve access and implement doula support should be considered as a strategy to decrease maternal mortality countywide in pursuit of the SDG targets. Additional studies to understand the impact of doula support on maternal and infant health outcomes in Brazil are needed. Furthermore, structural changes to the childbirth model of care must be made to ensure pregnant people can access doula support, to increase providers’ use of EBPs, and to promote a positive childbirth experience.

## Data Availability

The dataset supporting the conclusions of this article is available in the Dryad public repository (https://datadryad.org/stash); the current DOI of the data set is doi:10.5061/dryad.r7sqv9sb8.

## Abbreviations

EBP: evidence-based practices.
OR: odds ratio.
SDG: Sustainable Development Goals.
SoB: Senses of birth.
SUS: Brazilian Unified Health System.
WHO: World Health Organization.
